# Control of magnetite nanocrystal morphology in magnetotactic bacteria by regulation of *mms7* gene expression

**DOI:** 10.1038/srep29785

**Published:** 2016-07-15

**Authors:** Ayana Yamagishi, Masayoshi Tanaka, Jos J. M. Lenders, Jarla Thiesbrummel, Nico A. J. M. Sommerdijk, Tadashi Matsunaga, Atsushi Arakaki

**Affiliations:** 1 Division of Biotechnology and Life Science, Institute of Engineering, Tokyo University of Agriculture and Technology, Koganei, Tokyo, Japan; 2 Department of Chemical Science and Engineering, School of Materials and Chemical Technology, Tokyo Institute of Technology, Meguro-ku, Tokyo, Japan; 3 Laboratory of Materials and Interface Chemistry and TU/e Center of Multiscale Electron Microscopy, Department of Chemical Engineering and Chemistry, Eindhoven University of Technology, Eindhoven, the Netherlands; 4 Institute for Complex Molecular Systems, Eindhoven University of Technology, Eindhoven, the Netherlands

## Abstract

Living organisms can produce inorganic materials with unique structure and properties. The biomineralization process is of great interest as it forms a source of inspiration for the development of methods for production of diverse inorganic materials under mild conditions. Nonetheless, regulation of biomineralization is still a challenging task. Magnetotactic bacteria produce chains of a prokaryotic organelle comprising a membrane-enveloped single-crystal magnetite with species-specific morphology. Here, we describe regulation of magnetite biomineralization through controlled expression of the *mms7* gene, which plays key roles in the control of crystal growth and morphology of magnetite crystals in magnetotactic bacteria. Regulation of the expression level of Mms7 in bacterial cells enables switching of the crystal shape from dumbbell-like to spherical. The successful regulation of magnetite biomineralization opens the door to production of magnetite nanocrystals of desired size and morphology.

By means of organic molecules, organisms produce finely tuned inorganic materials, even nanoscale structures^1^,^2^,^3^,^4^. The relevant bioprocesses, collectively called biomineralization, are especially attractive to materials scientists because these processes also may facilitate the development of technologies for production of a wide variety of inorganic materials under mild conditions and in aqueous environments^5^,^6^,^7^,^8^,^9^,^10^,^11^. In recent comprehensive molecular studies, proteins and the corresponding genes that are involved in biomineralization have been characterized in various biological systems^12^,^13^,^14^. Genetically programmed expression of these proteins allows for highly regulated synthesis of materials in each organism^15^. Thus, by regulating gene expression of key molecules in a biological system, researchers can create a process for production of inorganic materials with desired structure and morphological features.

Magnetotactic bacteria synthesize a unique intracellular organelle, the magnetosome, composed of a magnetite (Fe_3_O_4_) or greigite (Fe_3_S_4_) crystal enveloped in a lipid membrane^16^. This biomineral has a size range of approximately 20–100 nm and a species-specific morphology, such as cuboctahedral, elongated prismatic, or bullet-shaped^17^,^18^,^19^. Given that magnetite nanocrystal-producing bacteria are prokaryotes, development of genetic modification protocols is relatively easy. In fact, the methods for transformation^20^, specific gene deletion^21^,^22^, and induction of target gene expression^23^,^24^ have already been developed for magnetotactic bacteria. The use of these approaches in proteomic and genetic studies^25^,^26^,^27^ to elucidate the molecular mechanisms of magnetite biomineralization helped to identify complex multistep pathways: localization of proteins^28^,^29^, invagination of the membrane^30^, vesicle formation^31^, actin filament formation^32^, assembly of vesicles onto the filament structure^33^, iron transport^34^, redox control^35^, crystal growth and morphological regulation^30^,^36^,^37^,^38^,^39^. In particular, identification of several proteins involved in the morphological regulation of magnetite crystal suggested that crystal morphology can be artificially controlled by use of these proteins. Previously, deficiency of the *mamS* or *mamT* gene resulted in the synthesis of small and elongated crystals^30^, and deletion of the *mms48* or *mms36* gene caused the production of large crystals^40^. However, the detailed functions of these proteins during the process of crystal morphological regulation is unclear.

Mms proteins (Mms5, Mms6, Mms7, and Mms13) have been identified as participants in the magnetite biomineralization process, and in this capacity, they are located on the surface of cuboctahedral crystals synthesized in *Magnetospirillum magneticum* strain AMB-1^36^. Several acidic amino acid residues that are found in the C-terminal regions of Mms proteins are thought to be involved in iron binding^41^,^42^ or stabilization of the magnetite crystal phase^43^. A peptide mimicking an Mms protein has been used to synthesize magnetic crystals *in vitro*^5^,^44^,^45^. Previously, the functions of the Mms proteins were evaluated by constructing gene deletion mutants. Although gene deletion may cause unexpected effects owing to a mutation within the genome or a polar effect on the expression of genes downstream of the target gene, analysis of a gene deletion mutant is nevertheless effective to reveal the *in vivo* function of a target protein. Deletion of the *mamGFDC* cluster including *mms7 (mamD*) and *mms13 (mamC*) was first performed in the *M. gryphiswaldense* MSR-1 strain, causing defects in crystal size^37^. Since the *mms13* gene deletion established in both the MSR-1 and AMB-1^46^ strains resulted in small cubo-octahedral crystals, Mms13 is involved in controlling crystal size. Deletion of the *mms6* gene resulted in small rod-shaped crystals^38^. Although a milder effect on crystal size and morphology was observed by deletion of the *mms6* gene in a previous study^39^, our work clearly shows that Mms6 is also involved in controlling crystal size and morphology. In addition, the *mms5* gene deletion mutant synthesizes small cuboctahedral crystals, as observed in the *mms13* gene deletion mutant, whereas the *mms7* gene deletion mutant synthesizes elongated rod-shaped crystals, as observed in the *mms6* gene deletion mutant^46^. According to these comparative data on characterization of gene deletion mutants, all *mms* genes are involved in the promotion of crystal growth^46^. The presence of two types of crystal shapes in the different *mms* gene deletion mutants suggests that Mms proteins have different effects on dimensions of crystal growth and that their coordinated functions determine the morphogenesis of magnetite nanocrystals^46^. These findings also imply that the crystal morphology can be controlled via regulation of the expression of genes encoding Mms proteins in a cell system.

The aim of the present study was the development of methods for control of magnetite biomineralization *in vivo*. For this purpose, we created an inducible *mms7* gene expression system in a mutant strain producing dumbbell-shaped crystals. Transmission electron microscopy (TEM) of these bacterial cells showed that the shape of the nano-sized magnetite biomineral crystals varies from dumbbell-like to spherical under the influence of different concentrations of a gene expression inducer. On the basis of these observations, we discuss the function of the Mms7 protein and morphogenesis of magnetite during biomineralization in magnetotactic bacteria. The genetic regulation of magnetite biomineralization opens the way not only to the elucidation of protein function but also for production of various magnetite particles with finely tuned magnetic properties. While different magnetite crystal morphologies have been found within specific magnetosome gene deletion mutants^30^,^35^,^37^,^39^,^47^, to our knowledge, this is the first report describing methods for the *in vivo* control of biomineral morphology by controlling gene expression.

## Results

### Characterization of a mutant strain synthesizing dumbbell-shaped crystals

**Figure 1:**
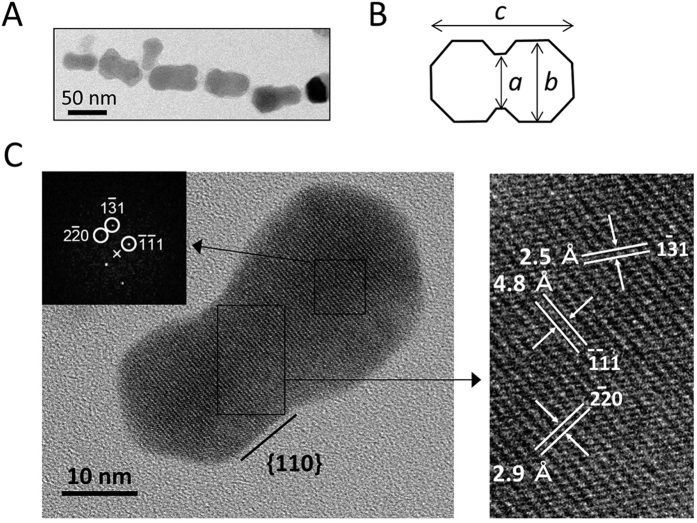
Characterization of magnetite crystals synthesized in the Δ*SID25* Δ*mms7* strain. Transmission electron micrographs of dumbbell-shaped crystals extracted from the Δ*SID25* Δ*mms7* strain (**A**) and drawing of a dumbbell-shaped crystal (**B**). A single dumbbell-shaped crystal was observed by high-resolution TEM (**C**). The black squares show corresponding FFT image and image at higher magnification, respectively.

In our previous study, a mutant strain of *M. magneticum* strain AMB-1 that synthesizes dumbbell-shaped crystals in cells was obtained when an *mms7* gene deletion mutant strain was established by homologous recombination (Fig. 1A)^46^. Based on genome sequence analysis, the *mms7* gene was replaced with a gentamicin resistance gene and an approximately 25 kbp region was spontaneously deleted from the MAI region in the mutant strain. This region was named the *SID25* (spontaneous internal deletion of 25 kbp) region^30^. Because the morphology of dumbbell-shaped crystals in *SID25* and the *mms7* gene deletion mutant strain (Δ*SID25* Δ*mms7*) has not been observed in other magnetotactic bacteria or chemically synthesized magnetite crystals, the characteristics of these dumbbell-shaped crystals extracted from the Δ*SID25* Δ*mms7* strain were analyzed by TEM. The average major axis *c* of dumbbell-shaped crystals (Fig. 1B) is 53.7 ± 7.0 nm long. The average lengths of the minor axes *a* and *b* (Fig. 1B) are 22.8 ± 3.8 and 31.4 ± 4.6 nm, respectively. High-resolution analysis shows a continuous crystal lattice for the dumbbell-shaped crystals (Fig. 1C). This feature clearly shows that the dumbbell-shaped crystals are not twinned but single crystals. The HRTEM image and its corresponding Fast Fourier Transform (FFT) image also indicate that the dumbbell-shaped crystals express {110} crystal faces (Fig. 1C). The lattice spacings observed for the crystals are consistent with the distances for the {111} faces, indicating that the crystals are elongated along the [111] direction.

### Induction of *mms7* gene by a tetracycline-inducible system

**Figure 2:**
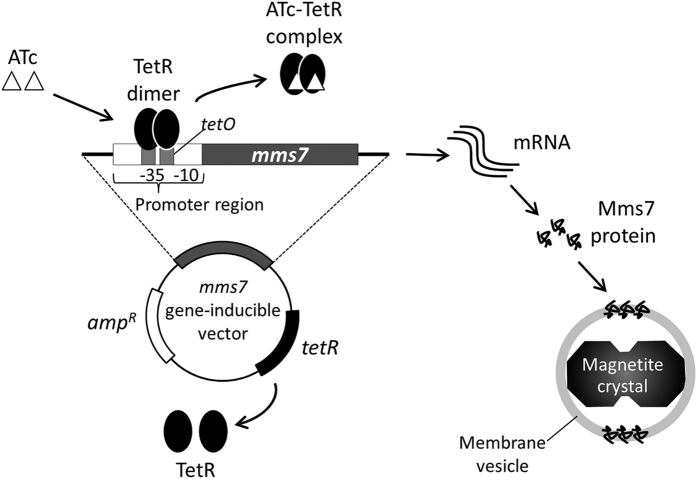
Schematic of tetracycline-inducible expression system. Inducible expression vector contains promoter harboring teracycline operator sequence (*tetO*), *mms7* gene, and tetracycline repressor gene (*tetR*). Two types of promoter, P_msp1_ and P_mms7_, are used for pUMtORM7 and pUMPmms7tORM7, respectively. Tetracycline repressor proteins expressed from inducible vector form a dimer and interacted to the *tetO* sequence contained in the promoter region. Inducer molecules (ATc) associate with the TetR dimer and induce the dissociation of dimer from *tetO* sequence.

A tetracycline-inducible expression system was used for regulation of *mms7* gene expression (Fig. 2). In this system, the target gene expression is induced by ATc, which binds to a repressor protein and triggers the dissociation of the repressor from the operator sequence (*tetO*) contained in the promoter region. By use of this system, a heterologous protein fused to the anchor Mms13 protein was successfully expressed on magnetite nanocrystals by the addition of an inducer to *M. magneticum* strain AMB-1^23^. Before the investigation, we confirmed the influence of ATc on the growth of the wild-type strain (Supplementary Fig. S1) and the formation of magnetite crystals (Supplementary Fig. S2). The results indicate that ATc concentrations between 0 and 2000 ng/mL have no significant effects on cell growth and magnetite formation.

To control the gene expression of *mms7*, we firstly used a pUMtOR plasmid, which contains a strong P_msp1_ promoter containing *tetO* sequences^23^. Based on the pUMtOR plasmid, an *mms7* gene-inducible expression vector (pUMtORM7) was constructed and transformed into the wild-type strain. In this study, ATc, as an inducer of *mms7* gene expression, was added to the medium when the cells were inoculated (final conc. 1.0 × 10^6^). The cells were cultivated for 48 h and then observed by TEM. Thus, the inducibly expressed Mms7 proteins affected newly synthesized crystals in the growing cells. Transformants cultured with ATc (0, 50, 100, 250, and 500 ng/mL) were analyzed by TEM (Supplementary Fig. S3). The results show that inducing the expression of the *mms7* gene does not affect crystal morphology in the wild-type strain. As the wild-type strain already expresses the native Mms7 protein, the bacteria may possess a regulatory mechanism to prevent expression at levels above what is necessary; alternatively, the overexpressed Mms7 protein may not properly function or localize onto magnetite crystals.

**Figure 3:**
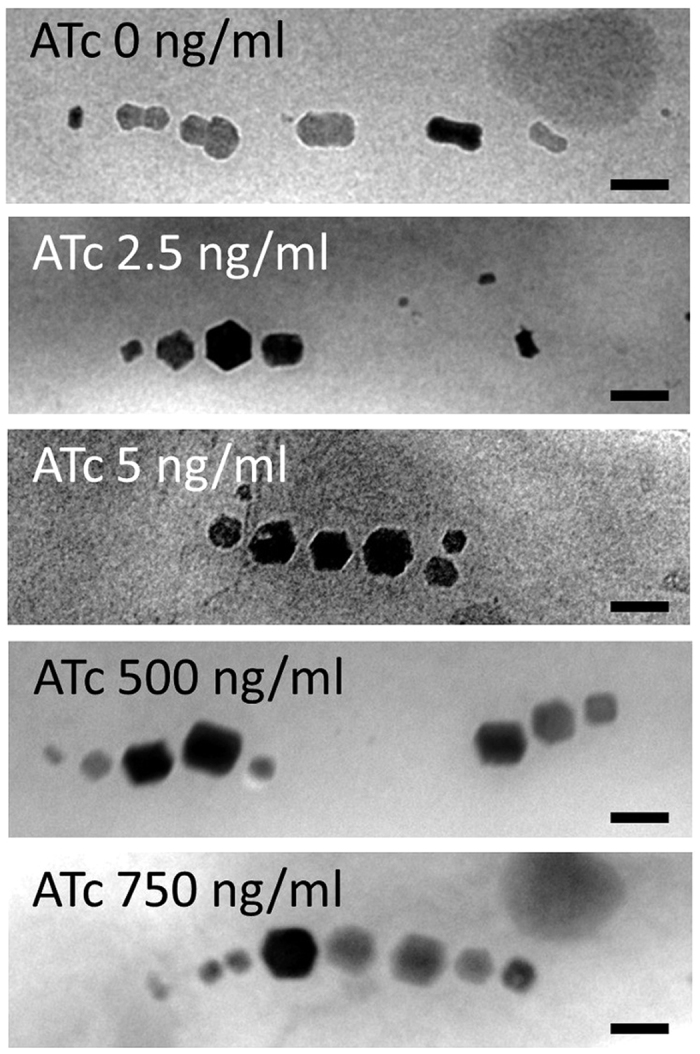
Transmission electron micrographs of magnetite crystals synthesized in the Δ*SID25* Δ*mms7* strain harboring pUMtORM7. The transformants were cultivated in the presence of 2.5–750 ng/mL ATc. Scale bar: 50 nm.

Next, the Δ*SID25* Δ*mms7* strain was used for the inducible expression system to avoid any effect of native Mms7 proteins on the magnetite crystals. Prior to the investigation of the effect of Mms7 protein expression induction on morphological regulation, the stability of the Δ*SID25* Δ*mms7* strain was evaluated over multiple cultivations. In order to suppress spontaneous mutations in the MAI region, the strain was cultured in the presence of 2.5 μg/mL gentamicin. After cultivating more than five times, stable dumbbell-shaped crystal production was observed. Using the Δ*SID25* Δ*mms7*–pUMtORM7 strain, the effect of Mms7 expression on morphological regulation was evaluated. In the presence of 500 ng/mL ATc, the Δ*SID25* Δ*mms7*–pUMtORM7 strain synthesized spherical crystals (shape factor: 0.85 ± 0.10), whereas the transformant cultured without ATc synthesized dumbbell-shaped crystals (shape factor: 0.62 ± 0.16) (Fig. 3) (Table 1), with a Mann-Whitney P-value < 0.05. The Mann-Whitney test is a non-parametric test for assessing whether two independent samples of observations come from the same distribution^48^. When the P-value is less than 0.05, the difference between the two analyzed samples is generally considered statistically significant^49^. Therefore, because the shape factors of crystals produced within cells grown in the presence of 0 and 500 ng/mL ATc were 0.62 ± 0.16 and 0.85 ± 0.10 (Mann-Whitney P-value < 0.05), respectively, the crystal morphology was significantly different.

In addition, the major and minor axes of synthesized crystals significantly decreased and increased, respectively, following the induction of *mms7* gene expression with 500 ng/mL of ATc (Mann-Whitney P-value < 0.05). In detail, the average lengths of the major and minor axes of the crystals in the Δ*SID25* Δ*mms7*–pUMtORM7 strain cultured with 500 ng/mL of ATc were 29.5 ± 11.2 and 25.0 ± 9.9 nm, respectively, and those without ATc were 36.6 ± 15.7 and 21.2 ± 7.6 nm, respectively (Table 1). These results indicate that the elongation of the major axis is suppressed and that of the minor axis is facilitated by the addition of ATc. We attribute the morphological changes in the magnetite crystals to the expression of the *mms7* gene in the Δ*SID25* Δ*mms7*–pUMtORM7 strain. Although various concentrations of ATc (2.5, 5, 500, and 750 ng/mL) were evaluated, no significant differences in crystal size and morphology were observed (data shown in Table 1 only reflect 500 and 750 ng/mL). This result suggests that the expression of the *mms7* gene is considerably induced even at lowest concentration of ATc, 2.5 ng /mL, and that the expressed Mms7 facilitates the growth of spherical crystals.

### Protein profile analysis of the *mms7* gene-inducing strain

**Figure 4:**
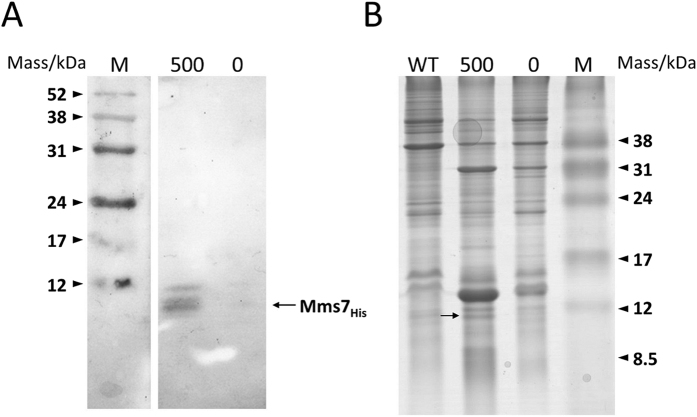
Analysis of Mms7 protein expression within magnetosomes. (**A**) Western blot analysis of proteins extracted from the magnetosomes synthesized in the Δ*SID25* Δ*mms7* strain harboring pUMPmms7tORM7 cultivated with 500 ng/mL ATc (lane 500) or without ATc (lane 0). M: Rainbow marker (low range). (**B**) SDS-PAGE analysis of magnetosome proteins in the wild-type (lane WT) and Δ*SID25* Δ*mms7* strain harboring pUMPmms7tORM7. The same samples as in (**A**) were loaded in the lanes labeled 500 and 0. M: Rainbow marker (low range). The black arrow shows the His-tag-fused Mms7 protein.

We attribute the morphological changes in the magnetite crystals following the addition of ATc to the action of the Mms7 protein, after its translation, on the magnetite crystals. To confirm Mms7 expression in magnetosome, the His-tag-fused Mms7 protein expression inducible vector pUMtORM7his was transformed into the Δ*SID25* Δ*mms7* strain. Based on the genomic and proteomic data from a previous study, the immature protein size was expected to be 30 kDa^25^, but the actual size of the mature Mms7 protein without the presumed N-terminus signal sequence was determined to be 7 kDa by its protein spot position in a two-dimensional electrophoresis gel image^36^. The results of the western blotting analysis of the magnetosome protein extracts from induced (500 ng/mL ATc) cell and non-induced (0 ng/mL ATc) cells confirmed the induced expression of His-tag-fused Mms7 at the expected band position (Fig. 4A). The multiple bands observed are probably due to post-translational modifications or degradation during sample preparation. In addition, from the comparison of the SDS-PAGE profiles, the amount of Mms7 protein in the induced cells was estimated to 1.3-fold higher than that in the wild-type strain (Fig. 4B). It is of note that the SDS-PAGE protein profiles are slightly different between these fractions. In the sample from induced cells, markedly lighter protein bands were found at approximately 15 and 30 kDa. As the western blotting results for the cell membrane fraction showed the presence of the His-tagged protein at around 30 kDa in addition to the band for the expected mature His-tag-fused Mms7 at 7 kDa (Supplementary Fig. S4), the band at 30 kDa might be immature Mms7 protein, although further investigation is required to clarify this conclusion.

### Shape control of magnetite crystal by regulation of *mms7* gene expression

Because the spherical-shaped crystals produced in the Δ*SID25* Δ*mms7*–pUMtORM7 strain were attributed to the high expression level of the *mms7*, the crystal morphology was expected to be regulated by a low expression of *mms7*. In the tetracycline-inducible expression system, the level of gene expression depends on the concentration of ATc, copy number of the plasmid, and intensity of the promoter. To control the expression over a wide range, the native *mms7* gene promoter (P_mms7_) harboring a tetracycline operator sequence was designed and used for constructing the inducible expression vector pUMPmms7tORM7. The induction of *mms7* gene expression in pUMPmms7tORM7 by the addition of ATc was evaluated by quantitative real-time PCR. The relative expression level of target mRNA in the Δ*SID25* Δ*mms7–*pUMPmms7tORM7 cultured in the presence of 50 ng/mL ATc was up to threefold that in the absence of ATc (Supplementary Fig. S5). The expression increased with an increase in the ATc concentration and was saturated at 250 ng/mL. In the presence of 250 or 500 ng/mL ATc, the level was as much as 4-fold that of the strain cultured without ATc. Thus, an inducible expression vector using the native promoter of the *mms7* gene can be used for regulation of expression level by the concentration of an inducer.

**Figure 5:**
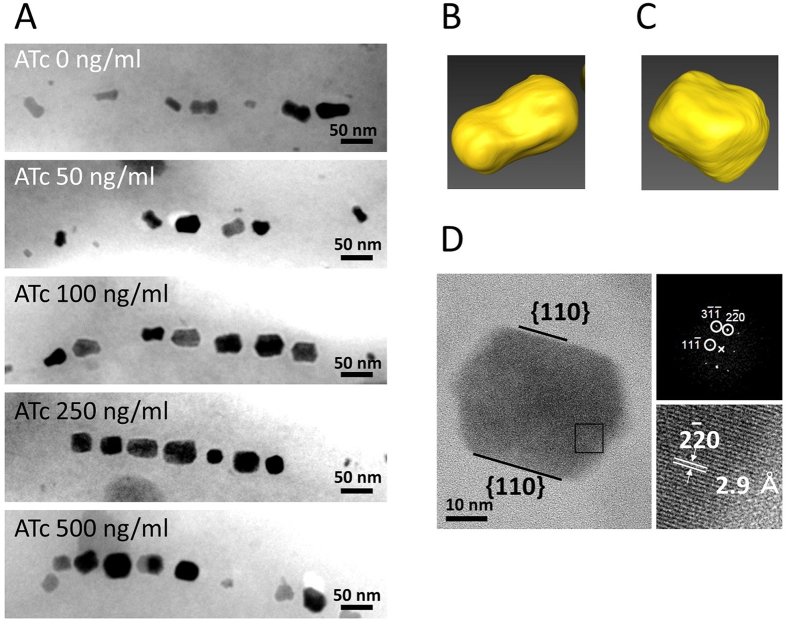
Magnetite crystals synthesized in the Δ*SID25* Δ*mms7* strain harboring pUMPmms7tORM7. The transformants were cultivated in the presence of 50–500 ng/mL ATc (**A**). 3D visualizations obtained by electron tomography for crystal extracted from transformants grown in the absence (**B**) or presence of 500 ng/mL ATc (**C**). HRTEM images of magnetite crystal synthesized in transformant cultured with 500 ng/mL ATc (**D**). The black square indicates the area used to obtain FFT pattern and image at higher magnification.

The transformant Δ*SID25* Δ*mms7–*pUMPmms7tORM7 cultured without ATc was observed by TEM and electron tomography (Fig. 5A,B, Supplementary Fig. S6, and Supplementary Movie S1). Based on these observations, the transformant synthesized dumbbell-shaped crystals whose minor axes *a* and *b* were similar to those in Δ*SID25* Δ*mms7* (Table 1). This result indicates that the transformation of pUMPmms7tORM7 did not affect the formation of magnetite crystals; thus, the leakage of *mms7* gene translation in the absence of ATc is insignificant. In the presence of 50–500 ng/mL ATc, the morphology of magnetite crystals synthesized in the Δ*SID25* Δ*mms7*–pUMPmms7tORM7 changed with the increase in ATc concentration (Fig. 5A). The strain cultured with 50–500 ng/mL ATc synthesized rod-shaped and spherical crystals (Fig. 5A). No definitive differences were observed between minor axes *a* and *b* in these crystals. When the strain was cultured with 250 and 500 ng/mL ATc, the frequency of spherical crystals (shape factor >0.8) was increased by approximately 40% in comparison with the result when the strain was cultured with 50 or 100 ng/mL ATc (approximately 10%). To assess the morphological changes of the magnetite crystals in the Δ*SID25* Δ*mms7*–pUMPmms7tORM7, the major and minor axis of the crystals were measured. In the Δ*SID25* Δ*mms7* strain harboring pUMPmms7tORM7, the minor axis of the crystal gradually but significantly increased when cultured under increasing concentrations of ATc from 0–250 ng/mL (0, 50, 100, and 250 ng/mL) (Mann-Whitney P-value <0.05), while in the case of 500 ng/mL, the values were not significantly different compared with those in the case of 250 ng/mL (Mann-Whitney P-value ≥ 0.05) (Supplementary Table S2). Although the average length of the major axis of crystals synthesized in Δ*SID25* Δ*mms7*–pUMPmms7tORM7 cultured under various concentrations of ATc is similar to that of the Δ*SID25* Δ*mms7* strain (Mann-Whitney P-value ≥ 0.05) (Supplementary Table S2), the average minor axis is elongated from 15.5 ± 6.5 nm to 25.1 ± 9.1 nm with increased concentration of ATc (Table 1). These results show that the change of crystal morphology from dumbbell- to spherical-shaped is due to the specific elongation of the minor axis (Table 1). As other possibilities, localization, expression level, or structural changes of other magnetosome proteins are considered to affect the morphological change of magnetite crystals.

In this study, 48 h after the induction of *mms7* gene expression, the morphology of magnetic particles within bacterial cells was observed by TEM. Therefore, the morphological change from a dumbbell-like structure to a spherical structure occurred within 48 h. It should be noted that no significant morphological change from the dumbbell-like structure was observed when the *mms7* gene was induced in the Δ*SID25* Δ*mms7* strain harboring pUMPmms7tORM7 when the cells were in the stationary phase, even after 24 h. This observation suggests that the tuning of magnetite production requires time for cell doubling to occur. However, a detailed time course experiment should be conducted in a future study to clarify the timescale for the morphological regulation using Mms7 protein in magnetotactic bacteria. We also performed gene induction experiments for the *mms7* gene several times, and the results indicated the repeatability of gene induction and crystal morphology regulation by ATc supplementation at different concentrations. However, we often observed unexpected gene mutations in the genome of the AMB-1 strain, as also reported by other researchers^30^,^49^. Thus, the Δ*SID25* Δ*mms7* strain was also kept at −80 °C as a glycerol stock.

In addition, the average number of crystals in the Δ*SID25* Δ*mms7*–pUMPmms7tORM7 cells cultured with 0–500 ng/mL of ATc is 19.4 ± 1.1. This result indicates that addition of ATc does not affect the number of crystals. In the presence of 500 ng/mL of ATc, electron tomography revealed that the shapes of magnetite crystals are clearly different from those in the absence of ATc (Fig. 5C, Supplementary Fig. S6, and Supplementary Movie S2). This result shows that the magnetite crystal morphology can be regulated by the *mms7* gene expression level. For further identification of the morphological characteristics, we analyzed the crystal faces of magnetite crystals in the *mms7* gene-inducible strain cultured with 500 ng/mL ATc by HRTEM. The magnetite crystals synthesized in the Δ*SID25* Δ*mms7* strain have dimple, and only express {110} crystal faces (Fig. 1C). In the Δ*SID25* Δ*mms7*–pUMPmms7tORM7 cultured with 500 ng/mL ATc, the crystals showed smoother {110} surfaces (Fig. 4D). Our previous study showed that the <111> crystallographic axes of the crystals in the wild-type and Δ*SID25* Δ*mms7* strains are parallel to the magnetosome chain alignment^46^. Based on the <111> crystallographic axes of crystals in Figs 1C and 5D, we propose that the {110} faces appear in the direction perpendicular to the magnetosome chain owing to the expression of the *mms7* gene.

## Discussion

In this study, we successfully controlled the morphology of magnetite crystals by regulating the expression of the *mms7* gene in bacterial cells. Because the size and shape of magnetic crystals directly influence their magnetic properties, the designer control over these factors is of great importance for the production of magnetic materials^50^. In the chemical synthesis of magnetic crystals, these factors are generally regulated by the addition of organic molecules, which function as a shape control agent to tune crystal growth^51^. Our results indicate that the growth rate along the minor axis of crystals can be regulated by simply changing the concentration of an external signal in the medium. Precise control over the specific growth direction in the range of 15–25 nm was achieved using a cellular system. The method introduced in this study can be used as an alternative way to synthesize magnetic crystals with a desired shape.

The magnetic properties are dependent on the size and morphology; thus, this tuning is very useful for various applications, including MRI and hyperthermia treatment^50^. By optimizing the culture conditions of a 10-L fermenter, the AMB-1 strain was found to produce 14.8 ± 0.5 mg dry weight/L of magnetite crystals over four days^52^. As reported previously, 1 mg of bacterial magnetite crystals is required for hyperthermia to treat cancer in a mouse model^53^, indicating that a high yield of tuned magnetite crystals for hyperthermia is expected to be achieved with large-scale culture and optimization of culture conditions. In addition, the biologically regulated production of magnetic nanoparticles in magnetotactic bacteria could have significant advantages over other synthetic routes such as imparting high-crystallinity, morphological definition, mono-dispersity, intrinsic biocompatibility, and biofunctionality with foreign protein display technologies, which makes this approach extremely attractive for nanotechnological applications, including medical diagnostics and therapies. Therefore, as the functional magnetic particles could be used in many different conditions, the tuning of magnetic properties in this system would be highly useful for the appropriate selection of magnetic particles with proper dispersibility for each condition.

When attempting to identify target gene function using a gene deletion technique, caution must be taken in the analysis because of potential unexpected effects, including a mutation at non-targeting genomic sites^49^ or a polar effect on genes located downstream of the target gene^39^, which can occur during the mutant strain establishment process. The recovery of a deficient phenotype by complementation of a deleted gene in the mutant strain provides meaningful evidence to show that the target gene is involved in the loss of function observed in the deletion mutant strain. Compared with the wild-type strain, small elongated crystals were synthesized in the *mms7* gene deletion mutant^46^. The observation is now complemented by an increase in both the minor axis and size of crystals in *mms7* gene-inducible strains. The stepwise increase in the minor axis, in spite of the constant major axis, is a strong indication that the Mms7 protein promotes crystal growth in a specific direction.

In bacterial magnetite biomineralization, several factors are considered to be influencing the magnetite crystal morphology, including the size of the reaction site^31^, the iron ion concentration^54^, and the redox potential in magnetosomes^35^. We previously showed that the size of membrane vesicles and iron uptake ability of the Δ*SID25* Δ*mms7* strain were similar to those of the wild-type strain, but the expression of magnetosome proteins differed^46^. This finding suggests that the protein components expressed in the membrane vesicle define the crystal size and morphology. In addition, as seen in the strain in which a high level of *mms7* gene expression was induced, elongation of the major axis is likely to be suppressed when an excessive amount of protein is expressed. Over-expressed Mms7 protein may act as an inhibitor of crystal growth.

Crystal surface analysis indicated that both dumbbell-shaped and spherical-shaped crystals formed with and without ATc have {110} crystal surfaces on the elongated side. This observation suggests that the expression of the Mms7 protein promotes crystal growth on the surface perpendicular to the elongation axis of dumbbell-shaped crystals. The crystal growth in this specific direction caused by the Mms7 protein is most probably related to the localization of the protein on the lateral side of the magnetosome vesicle perpendicular to the magnetosome chain axis^46^. The predicted structure of MamD protein, which is a homolog of Mms7 protein in AMB-1 strain, has a hydrophobic region in the N-terminal and a transmembrane helix in the C-terminal^55^. Mms7 protein identified from the surface of magnetite crystals has only a C-terminal helix region (7 kDa), which is cleaved from the premature Mms7 protein. Through its putative transmembrane helix region, Mms7 protein can localize in the magnetosome membrane, and the C-terminus is considered to be exposed to the inside of the magnetosome membrane vesicle. However, topology analysis of Mms7 protein in the magnetosome membrane is still required to elucidate Mms7 function.

The key region for crystal morphological regulation is thought to be the C-terminus due to the presence of acidic amino acids in this region. These acidic amino acid residues in the C-terminal region of the protein may also promote crystal growth by accumulating iron precursors such as ions or iron hydroxides. The protein may interact with the magnetite crystal to stabilize the growing crystal surface at the local site of the magnetosome vesicle. Although the iron binding ability of Mms7 has not been directly confirmed, a mimicking peptide of the Mms7 C-terminus was shown to be involved in the inhibition of green rust oxidation *in vitro*^43^. This result suggested that the C-terminus of Mms7 stabilizes the unstable crystal surface through an electrostatic interaction between the O atoms of the carboxyl groups in aspartic acid and glutamic acid and Fe^3+^ atoms^56^. Therefore, through the interaction between the Mms7 C-terminus and magnetite crystal surface, morphological regulation might occur during magnetite biomineralization in magnetotactic bacteria.

## Conclusions

In conclusion, morphological regulation of magnetite crystals was achieved by controlling of the expression level of the *mms7* gene in the Δ*SID25* Δ*mms7* strain. In the transformant harboring pUMPmms7tORM7, which induces a low expression of the *mms7* gene, crystal morphologies were switched from dumbbell-shaped to spherical. The size and minor axis of these crystals are elongated with increasing inducer concentration. To our knowledge, this is the first report of shape control of inorganic crystals by regulation of gene expression level in living organisms. By optimizing the culture conditions, the mass cultivation of cells can achieve a large amount of tuned magnetite crystals for various medical applications. A recent study of magnetosome reconstitution in non-magnetic cells showed expanded production of magnetite in bacterial cells^57^. Shape control of magnetite crystals by genetic regulation provides new opportunities for the *in vivo* synthesis of magnetite materials with controlled size, shape, and properties.

## Methods

### Strains and growth conditions

Strains, plasmids, and primers are described in detail in Supplementary Table S1. *Escherichia coli* strain TOP 10 (Invitrogen, CA, USA) was used for gene cloning. *E. coli* cells were cultured in LB medium at 37 °C after addition of 50 μg/mL ampicillin. *M. magneticum* AMB-1 (ATCC700264)^58^ was anaerobically grown in a glass vial or 10-liter fermenter using magnetic spirillum growth medium (MSGM). Colonies of *M. magneticum* AMB-1 were obtained on an MSGM plate that was incubated microaerobically at 28 °C, as described previously^38^. AMB-1 transformants by each vector were cultured under the same conditions in medium containing 5 μg/mL ampicillin. To induce *mms7* gene expression, the transformants were cultured in MSGM with 5–500 ng/mL anhydrotetracycline (ATc; Cole-Parmer Instrument, Vernon Hills, IL, USA).

### Size and crystallographic analyses of magnetite crystals by TEM

Low-magnification TEM analysis was performed using a conventional TEM (JEM1400, JEOL Ltd., Tokyo, Japan) at 100 kV. In this study, one major and two minor axes of at least 184 crystals were measured for each strain. The crystal size was evaluated as the average of major axis *c* and minor axis *a*, and the shape factor was calculated as minor axis *a* divided by major axis *c* (minor/major axis). Minor axes *a* and *b* in the crystal were measured for dumbbell-shaped crystals (Fig. 1B). HRTEM analysis was performed using TEM (H-9000NAR, Hitachi, Tokyo, Japan) at 300 kV, which has a point-to-point resolution of approximately 0.18 nm. The crystal faces were identified by indexing of the fast Fourier transform (FFT) patterns (JCPDS-International Centre for Diffraction Data, 2001). Using the symmetry of the planar distances in the FFT patterns, the spots of each FFT patterns were indexed.

### Magnetosome protein profile analysis by Tricine SDS-PAGE

Magnetosomes were extracted from cells cultured in 10 L medium. The cells were collected by centrifugation at 9,000 × *g* for 10 min at 4 °C and were then disrupted by passing them through a French press at 1,500 kg/cm^2^. The extracted magnetosomes were washed with 10 mM HEPES (pH 7.4) at least 10 times. The isolation of the cytoplasm–periplasm and cell membrane proteins was performed according to a previously described method^27^. The magnetosome protein fractions were extracted by boiling the magnetite crystals at 100 °C using a 1% SDS solution to allow the analysis of the total proteins on the magnetite crystal surface. The solution was sonicated for 10 sec every 10 min for 30 min to disperse the crystals. Protein concentrations were measured using the Pierce BCA Protein Assay Kit (Thermo Fisher Scientific, MA, USA) method with BSA as a standard. Tricine SDS-PAGE was performed according to the method described by Schagger^59^. Gels were stained with Bio-Safe Coomassie G-250 (Bio-Rad, CA, USA). The expression levels of Mms7 in each strain were measured by separating equal amounts of proteins and analyzing gel images using ImageQuant TL software (GE Healthcare, Little Chalfont, UK). Using the comparison between the wild-type strain and the *mms7*-inducible strain cultured with 500 ng/mL ATc, the expression level of Mms7 proteins was evaluated.

### SAED analysis and electron tomography (ET, 3D TEM)

For SAED analysis and electron tomography, 200 mesh Cu grids with continuous carbon films (Agar Scientific, Stansted, UK) were used. Sample preparation involved dropping 3 mL aqueous dispersion onto a TEM grid, blotting using filter paper and allowing the grid to dry in air. All TEM grids were surface plasma treated for 40 s using a Cressington 208 carbon coater prior to use. SAED analysis was performed on a FEI Tecnai 20 (type Sphera) operated at 200 kV, equipped with a LaB_6_ filament and a 1 k × 1 k Gatan CCD camera, and electron tomography was performed on the TU/e cryoTITAN (FEI, www.cryotem.nl) operated at 300 kV, equipped with a field emission gun (FEG), a postcolumn Gatan energy filter (GIF) and a post-GIF 2 k × 2 k Gatan CCD camera. Gatan DigitalMicrograph (including DiffTools) and ImageJ were used for TEM image and SAED pattern analysis.

The 3D reconstructions shown in Fig. 4, Supplementary Fig. S7, Supplementary Movie S1 and Supplementary Movie S2 were obtained from tilt series recorded using the following settings: tilt range: −64° to +64°, using constant 2° increments from 0° to +64° and to −64°; magnification: 19000x; pixel size: 0.4665 nm (0.92 nm after reconstruction); defocus: −500 nm; total dose: 100 e^−^A^−2^.

The alignment and 3D reconstruction of the raw data sets was performed with the software IMOD^60^. The segmentation and visualization of the 3D volumes was performed with the software Amira (Mercury Computer Systems) and Avizo (Visualization Science Group).

## Additional Information

**How to cite this article**: Yamagishi, A. *et al*. Control of magnetite nanocrystal morphology in magnetotactic bacteria by regulation of *mms7* gene expression. *Sci. Rep.* **6**, 29785; doi: 10.1038/srep29785 (2016).

## Supplementary Material

Supplementary Information

Supplementary Movie S1

Supplementary Movie S2

## Figures and Tables

**Table 1:** Statistical parameters of magnetite crystals from the *M. magneticum* AMB-1 wild type, the Δ*SID25* Δ*mms7* strain, and an *mms7* gene-inducible strain cultured under different concentrations of ATc.

**Strains**	**Plasmid**	**ATc concentration (ng/mL)**	**Crystal number/Cell**	**Major axis (nm)**	**Minor axis (nm)**	**Crystal size (nm)**	**Shape factor**	**Crystal morphology**	***n***
Wild type	–	0	18.3 ± 5.4	43.0 ± 14.0	39.4 ± 14.9	41.2 ± 14.4	0.91 ± 0.059	Spherical	220
Δ*SID*25 Δ*mms7*	–	0	19.7 ± 3.9	33.0 ± 12.0	14.1 ± 5.1	23.6 ± 8.1	0.45 ± 0.12	Dumbbell	197
Δ*SID*25 Δ*mms7*	pUMtORM7	0	18.5 ± 4.9	36.6 ± 15.7	21.2 ± 7.6	28.9 ± 11.2	0.62 ± 0.16	Dumbbell	185
Δ*SID*25 Δ*mms7*	pUMtORM7	500	11.5 ± 2.6	29.5 ± 11.2	25.0 ± 9.9	27.3 ± 10.4	0.85 ± 0.10	Spherical	115
Δ*SID*25 Δmms7	pUMtORM7	750	11.8 ± 3.1	30.0 ± 10.9	26.3 ± 10.2	28.2 ± 10.5	0.87 ± 0.09	Spherical	118
Δ*SID*25 Δ*mms7*	pUMPmms7tORM7	0	20.5 ± 5.1	30.4 ± 13.9	15.5 ± 6.5	23.0 ± 9.7	0.54 ± 0.15	Dumbbell	205
Δ*SID*25 Δ*mms7*	pUMPmms7tORM7	50	19.1 ± 4.3	29.9 ± 12.0	17.3 ± 7.5	23.6 ± 9.4	0.60 ± 0.15	Rod	191
Δ*SID*25 Δ*mms7*	pUMPmms7tORM7	100	18.4 ± 4.8	32.0 ± 12.1	20.0 ± 8.9	26.0 ± 10.1	0.63 ± 0.14	Rod	184
Δ*SID*25 Δ*mms7*	pUMPmms7tORM7	250	18.9 ± 5.8	32.6 ± 11.7	23.9 ± 9.6	28.2 ± 10.2	0.74 ± 0.15	Rod, Spherical	189
Δ*SID*25 Δ*mms7*	pUMPmms7tORM7	500	19.8 ± 3.4	33.1 ± 11.0	25.1 ± 9.1	29.2 ± 9.8	0.77 ± 0.13	Rod, Spherical	198

Data represents the mean ± standard deviation. Crystal size is the average of major and minor axes. Shape factor is calculated as minor axis divided by major axis (minor/major axis). At least 184 crystals were measured for each strain. Minor axis *a* in the crystals synthesized in the Δ*SID25* Δ*mms7* and Δ*SID25* Δ*mms7*–pUMPmms7tORM7 was used for statistical analysis.
