# Supplementary material for: Control of magnetite nanocrystal morphology in magnetotactic bacteria by regulation of mms7 gene expression
Source: Sci Rep. 2016 Jul 15;6:29785. doi: 10.1038/srep29785 (PMC4945951; doi:10.1038/srep29785)
Supplement: Supplementary Information [file srep29785-s1.pdf]

Supplementary Information

Control of magnetite nanocrystal morphology in magnetotactic bacteria by regulation of *mms7* gene expression

Ayana Yamagishi, Masayoshi Tanaka, Jos J. M. Lenders, Jarla Thiesbrummel, Nico A. J. M. Sommerdijk, Tadashi Matsunaga, *Atsushi Arakaki

Methods

Construction of mms7 gene-inducible expression vectors

For construction of pUMPmms7tORM7, the gene encoding the tetracycline repressor of *E. coli* (TetR) and an msp3 promoter (Pmsp3) was amplified as a template using the primers Msp3_F_EcoRI and TetR_R_EcoRI and pUMtOR [1]. These amplified fragments were digested and ligated in pUMG [2], generating the pUMR. The sequences 17 bp upstream of the putative 35, and the 10 promoter consensus sequences of Pmms7, which is located upstream of the *mamGFDC* operon, were replaced with tetracycline operator elements [3]. This Pmms7 derivative, named Pmms7^{tetO}, was amplified using the primers Pmms7(TetO)_F and Pmms7(TetO)_R_SspI, with the artificial synthetic DNA (TaKaRa) as a template. The PCR-amplified fragment of Pmms7(TetO) was cloned into the SspI site in pUMR, generating the pUMPmms7tOR. The *mms7* sequence was also amplified using the primers mms7_F and mms7_R, cloned into the SspI site in pUMPmms7tOR, generating pUMPmms7tORM7. For construction of pUMtORM7, the PCR-amplified *mms7* sequence was cloned into pUMtOR. Plasmids were transformed into wild-type *M. magneticum* AMB-1 by electroporation and a colony formation experiment was performed as previously described [2].

Quantitative real-time PCR

Total RNA was extracted from wild-type and *mms7* gene-inducible strains cultured at middle logarithmic growth phase, using Trizol reagent (Life Technologies). Genomic DNA was removed

from total RNA by PureLink® DNase Set (Life Technologies). RNA quality and quantity were checked by Agilent 2100 Bioanalyzer with the RNA 6000 Nano LabChip Kit. Total RNA (1 μg) was used to synthesize cDNA with a 1st strand Synthesis Kit (TaKaRa).

qRT-PCR was performed in a ViiA™7 Real-Time PCR System (Applied Biosystems), with Fast SYBR® Green Master Mix (Applied Biosystems) according to the manufacture instructions. Primer sets using qRT-PCR are shown in Table S1. cDNA samples of 1 μL were used as templates in 20 μL of PCR reagents.

Magnetosome protein profile analysis by Tricine SDS-PAGE

Isolation of the magnetosome, cytoplasm–periplasm, and cell membrane was performed [4]. The purified magnetosomes were boiled in 1% SDS solution to extract proteins from the surface of the magnetite crystals. Tricine SDS-PAGE was performed according to the method described by Schagger [5]. Gels were stained with Bio-Safe Coomassie G-250 (Bio-Rad). Expression levels of Mms7 and Mms13 were measured with ImageQuant TL software (GE Healthcare) and compared between the wild-type strain and the *mms7*-inducible strain cultured with 500 ng/mL ATc.

Western blotting analysis

For the determination of Mms7 protein expression in the gene-inducible strain, the Δ*SID25* Δ*mms7* harboring pUMtORM7his, which expresses His-tag-fused Mms7, was constructed. The

magnetosome protein fraction was extracted from this strain and analyzed by western blotting. The fraction was separated by Tricine SDS-PAGE and transferred to a polyvinylidene difluoride membrane. Target proteins were detected using His-tag with alkaline phosphatase (AP)-conjugated antipolyhistidine tag antibody (1:2000 dilution in 1% PBST), purchased from Sigma Aldrich Inc. 5-Bromo-4-chloro-3-indolyl phosphate/nitro blue tetrazolium (BCIP-NBT,Sigma) was used as the AP colorimetric substrate for visualization.

References

1 Yoshino, T., Shimojo, A., Maeda, Y. & Matsunaga, T. Inducible expression of transmembrane proteins on bacterial magnetic particles in *Magnetospirillum magneticum* AMB-1. *Appl Environ Microbiol* **76**, 1152-1157, doi:10.1128/AEM.01755-09 (2010).

2 Okamura, Y. *et al.* Design and application of a new cryptic-plasmid-based shuttle vector for *Magnetospirillum magneticum. Appl Environ Microbiol* **69**, 4274-4277 (2003).

3 Gatz, C. & Quail, P. H. Tn10-Encoded Tet-Repressor Can Regulate an Operator-Containing Plant Promoter. *Proceedings of the National Academy of Sciences of the United States of America* **85**, 1394-1397, doi:DOI 10.1073/pnas.85.5.1394 (1988).

4 Tanaka, M. *et al.* Origin of magnetosome membrane: proteomic analysis of magnetosome membrane and comparison with cytoplasmic membrane. *Proteomics* **6**, 5234-5247, doi:10.1002/pmic.200500887 (2006).

5 Schägger, H. Tricine-SDS-PAGE. *Nature protocols* **1**, 16-22, doi:10.1038/nprot.2006.4 (2006).

Supplementary Table S1 Bacterial strains, plasmids, and primers used in this study

Strains, plasmids, and primers	Description	Source or reference
Strains		
E. coli Top10	General cloning	Invitrogen
M. magneticum AMB-1	Wild-type strain	ATCC700264
mms7 and SID25 region deletion mutant	*mms7* gene and SID25 region deletion mutant strain	Arakaki et al. (2014)
Plasmids		
pUMG	BamHI-digested whole fragment of pMGT cloned in pUC19;Amp^r	Okamura et al. (2003)
pUMtOR	Template to PCR-amplify *Pmsp3-TetR*	Yoshino et al. (2010)
pMD20TP7tetO	Template to PCR-amplify *Pmms7(TetO)*	TaKaRa
pUMtORM7	pUMG with *Pmsp3-TetR* and *Pmsp1(TetO)-mms7*	This study
pUMtORM7his	pUMG with *Pmsp3-TetR* and *Pmsp1(TetO)-mms7his*	This study
pUMPmms7tORM7	pUMG with *Pmsp3-TetR* and *Pmms7(TetO)-mms7*	This study
*Primers**		
Msp3_F_EcoRI	5′ - TTTTTT*gaattc*ACGTTGAATCCCAGCGCCC -3′	This study
TetR_R_EcoRI	5′- CCCCCC*gaattc*TTAAGACCCACTTTCACATTTAAG -3′	This study
Pmms7(TetO)_F	5′- CAACCCCATCCGGTTTGGCG -3′	This study
Pmms7(TetO)_R_SspI	5′- *aatatt*GACTCTACACTCCGAATCCTC -3′	This study
mms7_F	5′- ATGCAGGACCTTCTCCTGGCCAAG -3′	This study
mms7_R	5′- TTATTCTTCCCCGGCCGCAG -3′	This study
mms7His_R	5′- TTAGTGATGATGGTGGTGATGTTCTTCCCCGGCCGCAG -3′	This study
Pmms7_F_HindIII	5′- TCTCTC*aagctt*ACGACAGCGGAGAAGTTGCG -3′	This study
Pmms7_R_SpeI_HindIII	5′- GAGAGA*aagcttactagt*GACTCTACACTCCGAATCCTCACGGA GAGAAAGCTTACTAGTGACTCTA CACTCCGAATCCTCACG -3′	This study
mms7_F_Spe I	5′- TCTCTC*actagt*ATGCAGGACCTTCTCCTGGCCAAG -3′	This study
mms7_R_SpeI	5′- TCTCTCactagtTTATTCTTCCCCGGCCGCAG -3′	This study
rpoA_F	5′- CCAGCTCTTCGATGTTCTCC -3′	This study
rpoA_R	5′- CAACTGCCTGAAGAACGACA -3′	This study
RTmms7_F	5′- CCTTCTCCTGGCCAAGGTC -3′	This study
RTmms7_R	5′- ATGGTGGCGAGATTGGTG -3′	This study

*Restriction enzyme sites are indicated by small italicized characters.

Supplementary Table S2. Statistical analysis of magnetite crystals from Δ*SID25* Δ*mms7* – pUMPmms7tORM7 cultured with various concentrations of ATc

Strain	ATc concentration (ng/mL)	Major axis Mean ± SD (nm)	Major axis P-value	Minor axis Mean ± SD (nm)	Minor axis P-value	Shape factor Mean ± SD (nm)	Shape factor P-value	*n*
Δ*SID25* Δ*mms7* – pUMPmms7tORM7	0	30.4 ± 13.9	0.92	15.5 ± 6.5	1.9×10^{-2}*	0.54 ± 0.15	4.9×10^{-2}*	205
	50	29.9 ± 12.0		17.3 ± 7.5		0.60 ± 0.15		191
	50	29.9 ± 12.0	0.15	17.3 ± 7.5	5.7×10^{-3}*	0.60 ± 0.15	4.4×10^{-3}*	191
	100	32.0 ± 12.1		20.0 ± 8.9		0.63 ± 0.14		184
	100	32.0 ± 12.1	0.18	20.0 ± 8.9	1.2×10^{-6}*	0.63 ± 0.14	8.2×10^{-7}*	184
	250	32.6 ± 11.7		23.9 ± 9.6		0.74 ± 0.15		189
	250	32.6 ± 11.7	0.56	23.9 ± 9.6	0.80	0.74 ± 0.15	1.6×10^{-3}*	189
	500	33.1 ± 11.0		25.1 ± 9.1		0.77 ± 0.13		198

Data represent the mean ± standard deviation. The shape factor was calculated as the minor axis divided by the major axis (minor/major axis). At least 184 crystals were measured for each strain. Minor axis *a* in the crystals synthesized in the Δ*SID25* Δ*mms7*–pUMPmms7tORM7 mutant was used for statistical analysis. The Mann-Whitney test was used to acquire P-values. *P < 0.05

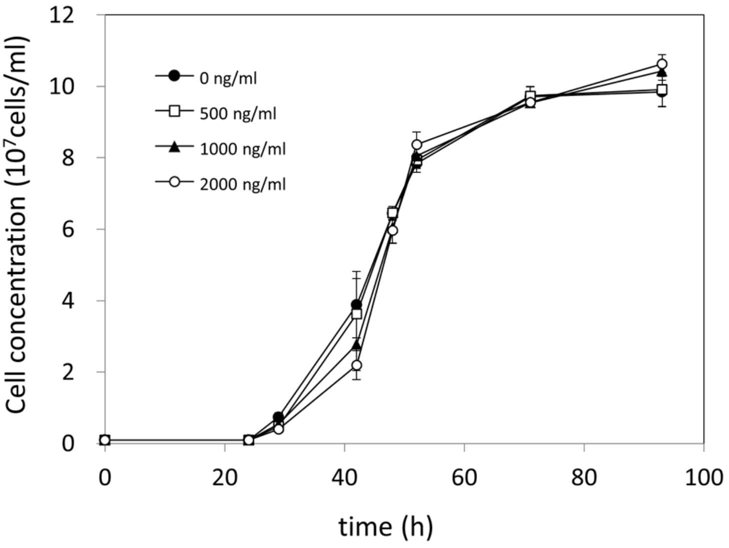

Supplementary Fig. S1 Growth curve of the AMB-1 wild-type strain in the presence of ATc. Cells were cultivated in MSGM under different concentrations of ATc (0, 500, 1000, and 2000 ng/mL). Addition of 0–2000 ATc has no influence on the growth of AMB-1 wild-type strain.

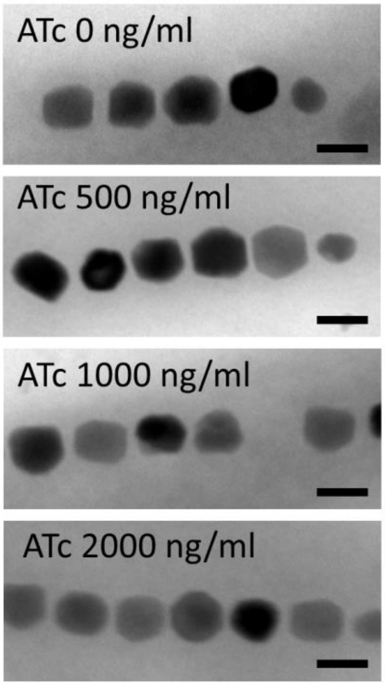

Supplementary Fig. S2 Transmission electron micrographs of magnetite crystals synthesized in the wild-type strain cultivated in the presence of 500–2000 ng/ml ATc. ATc concentrations 0–2000 ng/mL have no significant effects on magnetite formation. Scale bar: 50 nm.

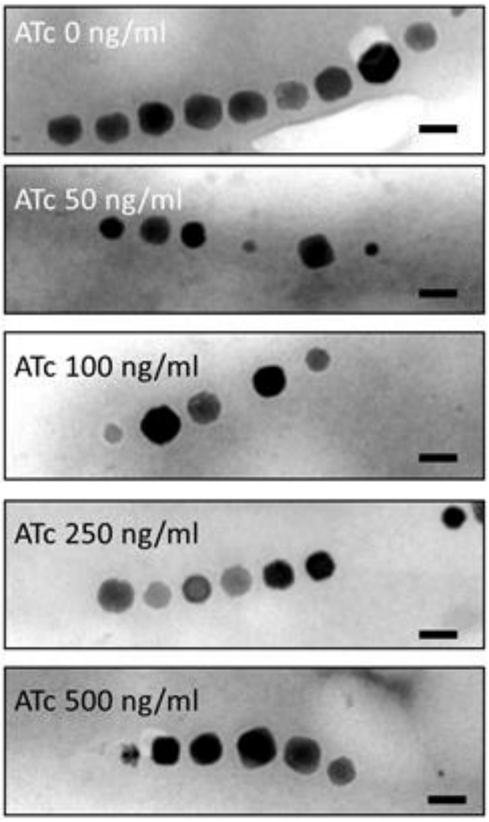

Supplementary Fig. S3 Transmission electron micrographs of magnetite crystals synthesized in a wild-type strain harboring pUMtORM7. The transformants were cultivated in the presence of 2.5–750 ng/ml ATc. Induction of *mms7* gene expression did not affect crystal size and morphology. Scale bar: 50 nm.

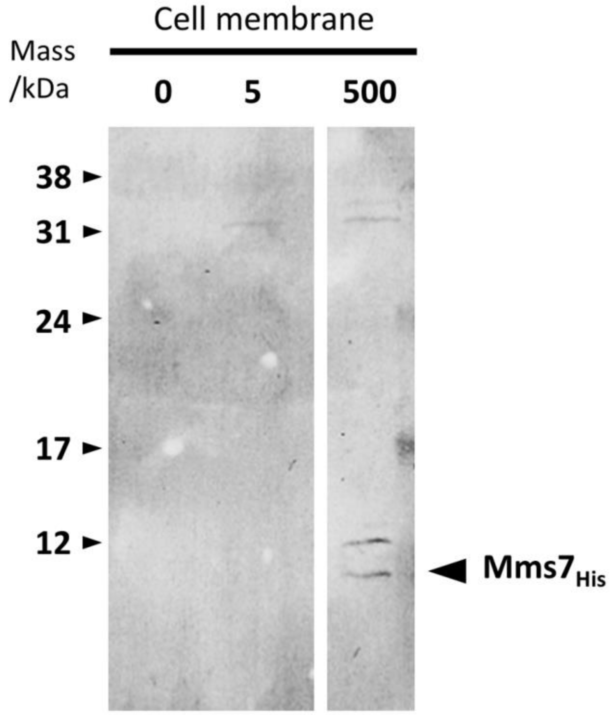

Supplementary Fig. S4 Western-blotting analysis of cell membrane fraction extracted from Δ*SID25* Δ*mms7*–pUMtORM7his. Cells were cultured in the absence or presence of ATc (5 or 500 ng/mL). Proteins were detected by an AP-conjugated anti-His tag antibody. Black arrowhead indicated His-tag-fused Mms7 protein.

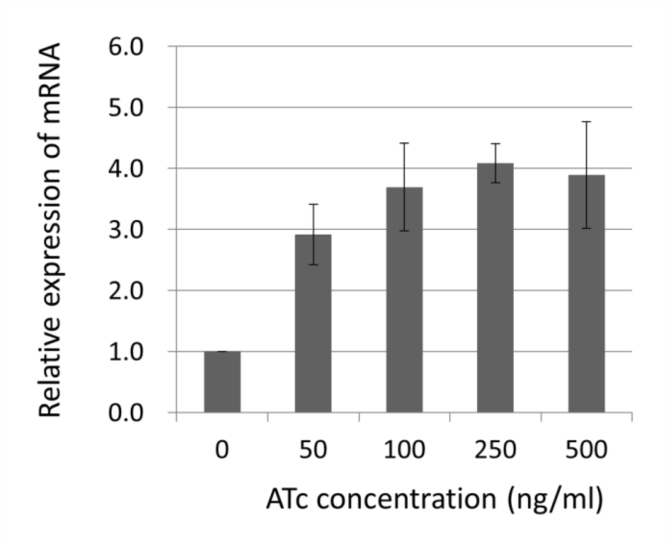

Supplementary Fig. S5 Relative expression level of mRNA of the *mms7* gene in the Δ*SID25* Δ*mms7*-harboring pUMPmms7tORM7 strain cultured with ATc (0, 50, 100, 250, and 500 ng/mL).

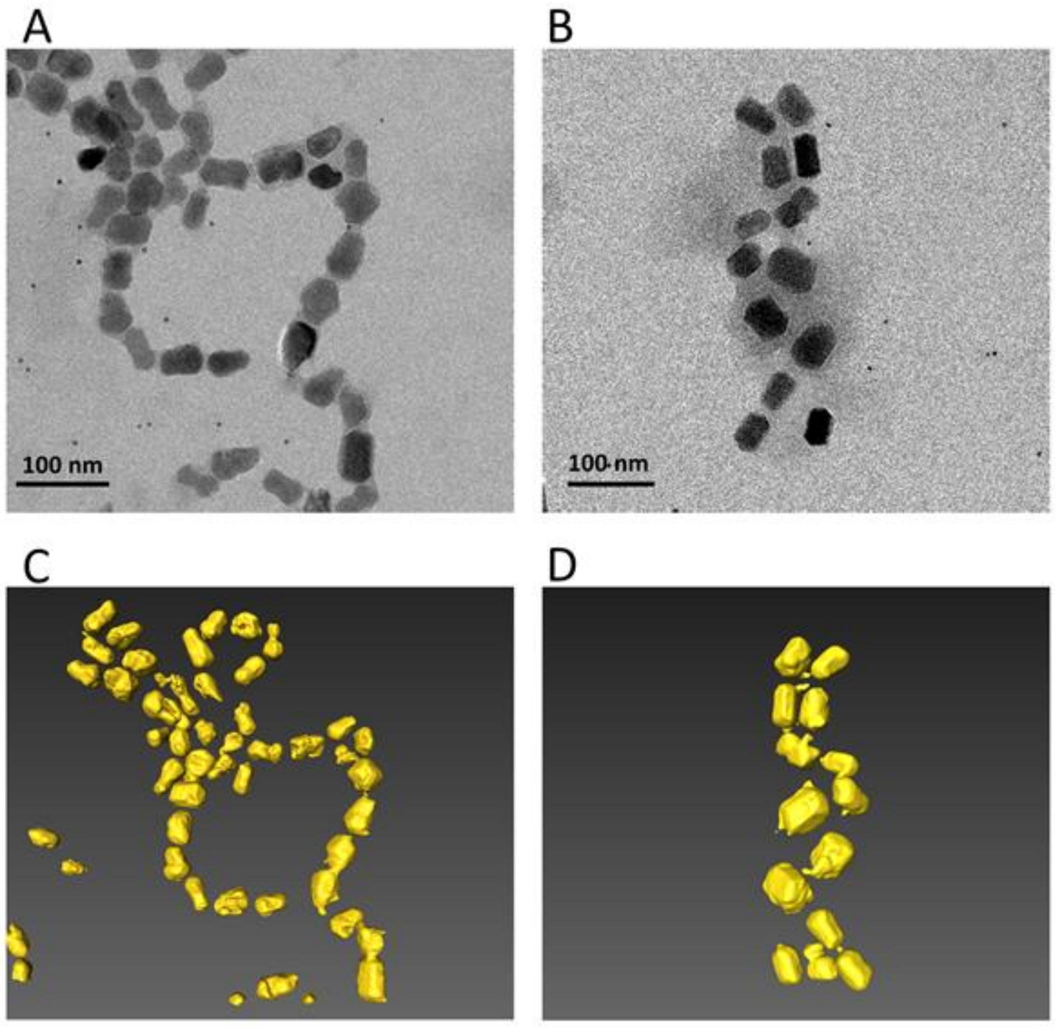

Supplementary Fig. S6 Transmission electron micrographs (A and B) and overview of 3D reconstructed images (C and D) of magnetite crystals synthesized in the Δ*SID25* Δ*mms7* strain harboring pUMPmms7tORM7 cultured in the absence (A and C) or presence of 500 ng/ml ATc (B and D). These crystals were observed by electron tomography.

Supplementary Movie S1 Electron tomography reconstruction of magnetite crystals extracted from the Δ*SID25* Δ*mms7*-harboring pUMPmms7tORM7 strain cultured without ATc

Supplementary Movie S2 Electron tomography reconstruction of magnetite crystals extracted from the Δ*SID25* Δ*mms7*-harboring pUMPmms7tORM7 strain cultured with 500 ng/ml of ATc
